# The Brazilian Hereditary Cancer Network: historical aspects and challenges for clinical cancer genetics in the public health care system in Brazil

**DOI:** 10.1590/1678-4685-GMB-2014-0373

**Published:** 2016-06-03

**Authors:** Patricia Ashton-Prolla, Hector N. Seuanez

**Affiliations:** 1 Departamento de Genética, Universidade Federal do Rio Grande do Sul and Serviços de Pesquisa Experimental e Genética Médica, Hospital de Clínicas de Porto Alegre, Porto Alegre, RS, Brazil; 2 Departamento de Genética, Universidade Federal do Rio de Janeiro and Divisão de Genética, Instituto Nacional de Câncer (INCA), Rio de Janeiro, RJ, Brazil

Although the majority of cancers result from complex interactions between genetic, epigenetic and environmental factors, inherited germ-line mutations with high penetrance account for approximately 5-10% of all tumors (Weitzel *et al.*, 2010). Extrapolating these estimates to the Brazilian population, 2,850 to 5,720 of the 57,120 new breast cancer and 1,630 to 3,260 of the 32,600 new colorectal tumors are expected to be hereditary according to 2014 estimates (http://www.inca.gov.br/estimativa/2014/).

During the last two decades our understanding of the molecular mechanisms of cancer has immensely increased. New technologies like new generation sequencing (NGS) and in-depth analyses of large tumor series (as the ones included in The Cancer Genome Atlas - TCGA) have identified driver and associated mutations in most tumors and provided significant insights on the molecular pathways of the different stages of carcinogenesis (Ciriello *et al.*, 2013; Liu and Zhang 2014). In the field of clinical cancer genetics, several genes directly involved in hereditary cancers have been identified and characterized and more than 50 syndromes associated to cancer predisposition have been described to date (Hodgson and Maher, 1993; Lindor *et al.*, 2008). These discoveries led to the development of programs for Genetic Cancer Risk Assessment (GCRA) for identifying and managing patients and families with hereditary cancer. GCRA has been defined as "a specialized clinical practice that requires knowledge of genetics, oncology and patient and family counseling skills involving more provider time than most other clinical services" (Weitzel *et al.*, 2011).

GCRA programs are characteristically carried out in academic and tertiary care health centers with multidisciplinary teams providing diagnosis, genetic counseling, and frequently, long term management of patients and their relatives (MacDonald *et al.*, 2010). Several guidelines have been recommended by professional societies in North America and Europe, like the American Society of Clinical Oncology (ASCO), the European Society of Medical Oncology (ESMO), the European Society of Human Genetics (ESHG), the National Society of Genetic Counselors (NSGC), the Oncology Nursing Society (ONS) and other health care professional organizations (like the EuroGenTest). These guidelines have outlined the basic standards for cancer risk counseling, risk assessment and genetic testing (American Society of Clinical Oncology, 2003; Trepanier *et al.*, 2004; Dierking *et al.*, 2013; Stoffel *et al.*, 2014). More recently, the reduced costs of genetic tests, the possibility of multiplex gene assays and NGS have significantly improved access to GCRA programs. However, several challenges and limitations still persist for a successful, molecular diagnosis of hereditary cancer, like ethical aspects arising from unsolicited or unexpected results or absence of identifiable mutations in a significant proportion of families with hereditary cancer phenotypes (the "missing heredity" phenomenon, observed in 40-60% of families, depending on the phenotype) (Samuel *et al.*, 2014; Tung *et al.*, 2015).

In view of the complexity involved in hereditary cancer care, the National Cancer Institute (NCI) of the United States of America proposed, in 1996, the launching of a national, cancer genetics network as a collaborative venture involving investigators from different institutions (Jenks, 1996). The Cancer Genetics Network (CGN) was officially announced in September 1998, comprising eight specialized centers focused on the study of hereditary predisposition to cancer and supported by a US$ 5.8 million grant for five years and a US$ 1.28 million grant for Information Technology and Informatics. This network offered support for collaborative studies on the hereditary basis of cancer susceptibility and for incorporating new knowledge into the daily medical practice and to the ethical, legal and social implications (ELSI) involved in assessing hereditary cancer risk. All these centers were engaged in bio-banking activities and offered genetic counseling to patients and their families, as well as educational programs to health care professionals. To date, the CGN congregates 14 centers and 26,000 registered participants (http://www.cancergen.org/). In addition to these academic models providing GCRA, additional models have been proposed over the years for extending access to the clinical practice, including a community-of-practice model that leverages the experience and multidisciplinary nature of academic programs provided by collaborations of academic centers with community-based providers in practice networks (Allain *et al.*, 2010; Weitzel *et al.*, 2011).

The development of Clinical Cancer Genetics in Brazil over the past twenty years provides an interesting model for implementing comprehensive strategies in other underdeveloped countries. In the mid-90s, several centers organized specialized clinics for assessing hereditary cancer risk, as well as for diagnosing and managing patients and families with hereditary cancer syndromes. These initiatives arose from the most advanced academic and research centers of public health in large cities of the Southeast of Brazil, and were supported by research programs for generating expertise in genetic testing. Standards for counseling, surveillance, testing and care were modeled on highly-structured programs in North America and Western Europe, involving multidisciplinary teams of specialized medical doctors, nurses and psychologists (Palmero *et al.*, 2007). These teams developed practices adapted to the cultural and socio-economic diversity of the populations that these programs were devised to assist. Genetic counseling and surveillance encompass the long-term follow-up of patients and families, as well as the development of specific programs for increasing awareness and early detection.

Despite these advances, the current coverage of oncogenetic services is presently restricted to less than 5% of the Brazilian population. In fact, the vast majority of the population relies on the Public Health Care System (Sistema Único de Saúde, SUS) whose policies and strategies to identify and care for patients and families with hereditary cancer are still unsatisfactory. To address this issue, the Brazilian National Cancer Institute (INCA, Instituto Nacional de Câncer) convened the Brazilian Hereditary Cancer Network (BHCN), initially supported by public funding by the National Council for Scientific and Technical Development (CNPq). Several research projects were initially implemented for generating expertise in genetic counseling and testing protocols for the most common cancer susceptibility syndromes. In addition, specialized training programs for MDs and other health care professionals involved in hereditary cancer were implemented by BHCN participating centers. In 2009, a standard handbook was published for establishing nationwide guidelines for the detection, diagnosis, counseling, testing and surveillance of hereditary cancer syndromes (http://www1.inca.gov.br/inca/Arquivos/publicacoes/Cancer_Familial_fim.pdf). In 2011, the BHCN was consolidated by establishing reference centers for research and clinical care of hereditary cancer. Currently, ten centers are established in public hospitals in cities like Belém (in the northern region and the Amazon basin), Salvador (in the northeastern region), Vitória, Rio de Janeiro, São Paulo, Ribeirão Preto, Barretos (in the southeastern region) and Porto Alegre (in southern Brazil), altogether providing approximately 7,000 outpatient consultations and risk assessments of hereditary cancer per year to the population assisted by the Public Health Care System (SUS) of Brazil. However, this coverage is still limited because these centers are located in urban areas that do not include large sections of the rural population and because their efficacy is constrained by shortage of properly trained, medical and non-medical, health care professionals and also by the fact that SUS does not pay for the genetic test to confirm cancer predisposition. Unlike most European and North American centers, genetic counseling in Brazil is mostly provided by physicians (MDs) because genetic counseling *per se* is not yet recognized as a profession. Moreover, as SUS does not contemplate financial support for genetic testing for hereditary cancer syndromes, the cost of most assays has been partially covered by funds originally destined for research rather than clinical assistance. A significant advance, however, took place in 2012, when the coverage of genetic testing by private health care plans became mandatory in Brazil, currently covering around 20-30% of the population.

Major challenges lay still ahead for the Brazilian Hereditary Cancer Network for facilitating access of individuals with suspected or confirmed hereditary cancer to the public health care system for genetic testing, screening, and health management. These challenges require recognizing the relevance of genetic counseling, testing and inclusion of genetic tests in SUS, developing initiatives for supporting institutions currently providing clinical care in cancer genetics, dissemination of information to remote areas of the country, and creation of training programs and career opportunities for health care professionals in cancer genetics. Moreover, incorporation of novel diagnostic technologies, multidisciplinary approaches to patient care and establishment of collaborative research projects must be stimulated, aiming at a more comprehensive understanding of hereditary cancer in Brazil.
